# Development of a quantitative method to measure EV uptake

**DOI:** 10.1038/s41598-019-47023-9

**Published:** 2019-07-19

**Authors:** Víctor Toribio, Sara Morales, Soraya López-Martín, Beatriz Cardeñes, Carlos Cabañas, María Yáñez-Mó

**Affiliations:** 1 Departamento de Biología Molecular, Universidad Autónoma de Madrid (UAM), Madrid, Spain; 2 Instituto de Investigación Sanitaria La Princesa (IIS-IP), Madrid, Spain; 3 Centro de Biología Molecular Severo Ochoa CSIC-UAM, Madrid, Spain; 4 Departamento de Inmunología, Oftalmología y OTR (IO2), Facultad de Medicina, Universidad Complutense, Madrid, Spain; 5 Instituto de Investigación Sanitaria Hospital 12 de Octubre (i+12), Madrid, Spain

**Keywords:** Cell biology, Biological techniques

## Abstract

The outstanding potential of Extracellular Vesicles (EVs) in medicine, deserves a detailed study of the molecular aspects regulating their incorporation into target cells. However, because EV size lies below the limit of resolution of optical techniques, quantification together with discrimination between EV binding to the target cell and uptake is usually not completely achieved with current techniques. Human tetraspanins CD9 and CD63 were fused to a dual EGFP-Renilla-split tag. Subcellular localization and incorporation of these fusion proteins into EVs was assessed by western-blot and fluorescence microscopy. EV binding and uptake was measured using either a classical Renilla substrate or a cytopermeable one. Incubation of target cells expressing DSP2 with EVs containing the complementary DSP1 portion could not recover fluorescence or luciferase activity. However, using EVs carrying the fully reconstituted Dual-EGFP-Renilla protein and the cytopermeable Renilla luciferase substrate, we could distinguish EV binding from uptake. We provide proof of concept of the system by analysing the effect of different chemical inhibitors, demonstrating that this method is highly sensitive and quantitative, allowing a dynamic follow-up in a high-throughput scheme to unravel the molecular mechanisms of EV uptake in different biological systems.

## Introduction

Extracellular Vesicles (EVs) are a highly heterogeneous group of biological nanoparticles, which can be secreted by almost every living organism and can be isolated from any biological fluid^1^. EVs are encapsulated by a lipidic bilayer membrane that incorporates specific glycoproteins and transmembrane proteins, among which different tetraspanin members are highly represented, together with selective luminal components, including proteins, RNAs and metabolites^1^. The different existing types of EVs are commonly classified in relation to their biogenesis pathway, which dictates some characteristics of their cargo as well as biophysical properties^1,2^. Thus, EVs are divided into two main groups: exosomes, generated at the endosomal pathway by inward budding of the membrane of multivesicular bodies (MVBs) and subsequently released by fusion of these MVBs with the plasma membrane^3^ and microvesicles, which are produced by the evagination of the plasma membrane^4^. A common feature of EVs is that they play an essential role in intercellular communication and signalling, at both paracrine and endocrine levels^1^. Thus, their physiological functions are highly diverse, comprising from immune response regulation^5^, reproduction^6^, development, to tissue repair^7,8^. EV composition directly depends on the producer cell type and its metabolic state, so they also play different roles in pathologic conditions, like cancer progression^9^ or Alzheimer’s disease^10^. In addition, EVs have been suggested to have a very promising therapeutic potential. For example, EVs secreted from mesenchymal stem cells (MSCs) are able to protect against ischemia and induce tissue regeneration^7,11^. The use of EVs from parasites can also regulate immune function in the treatment of inflammatory diseases^12,13^.

However, although some effects of EVs on target cells may be elicited by binding to surface receptors and triggering intracellular signalling cascades, most of these effects are thought to be mediated by EV uptake. EV uptake pathways can be as heterogeneous as EV composition and origin. Different pathways have been reported to mediate their internalization or uptake: both clathrin-and caveolin-mediated endocytosis^14–16^ micropinocytosis^17,18^, phagocytosis^19^, internalization through lipid rafts^20^, lectins^21^ and even cell surface membrane fusion^22^. Each different cell type may use different EV uptake pathways but generally EV uptake seems to be a complex and cooperative process involving a combination of different pathways^23^. However, although some preliminary evidence shows a heterogeneous landscape, EV uptake has not been thoroughly studied and it is still a field that deserves further research to unravel the key molecular mechanisms involved in this essential process.

Several techniques for studying EV uptake have been developed. The majority of them are based on staining EVs with fluorescence or luminescence in order to detect and track them during EV uptake process. Some of the probes used are membrane-specific fluorescent dyes, such as PKH67, PKH26, DiI, DiR, or rhodamine B^24^. However, these dyes may affect normal EV behaviour, they have a short half-life and can easily give non-specific signal in target cells by direct exchange of the dye between both membranes, since they stain lipids that may be present in the membranes of the samples, and can produce false EV signals by forming micelles^25^. In addition, they are not useful to distinguish between different EV subpopulations^24^. Other fluorescent labelling strategies directed to EV proteins have already been described^26^. Some studies have made use of reporter proteins targeted to EVs by using a palmitoylation signal^27^. Alternatively, fluorescent reporter proteins have been fused to EV markers like the tetraspanin CD63^28,29^. These strategies allow to label EVs from their early biogenesis and to track them during their release or uptake. Moreover, they have a longer half-life, thus allowing to perform *in vivo* EV tracking experiments. EVs carrying luciferase activity have also been developed, mainly for *in vivo* EV uptake imaging^30–32^. However, since EV size is under the resolution limit of most optical techniques, all these techniques fail to quantitate EV uptake, since a cluster of EVs could render a single spot in the image. To clearly discriminate between EV attachment to the cell surface and their uptake detailed confocal imaging is required, hampering the study of the molecular mechanisms involved in the latter process.

Thus, in this report we aimed at developing a new quantitative and highly sensitive EV uptake assay. Our proposed assay is based on a pair of chimeric reporter proteins, DSP1 (Dual Split Protein) and DSP2. DSP1 is formed by aminoacids 1 to 229 of Renilla luciferase and β-sheets 1 to 7 of EGFP, whereas DSP2 encompasses aminoacids 230 to 311 of Renilla luciferase and β-sheets 8 to 11 of EGFP^33^. DSP1 and DSP2 are able to self-reassociate when they are present in the same compartment, thus recovering both the green fluorescence and luciferase activities. This strategy has been previously used to study cell fusion induced by viral infection or viral entry in target cells^34,35^. We generated DSP1-tagged tetraspanin constructs to specifically direct them into EVs and report the efficacy of different experimental approaches to develop a highly sensitive method to quantify EV uptake. This assay will allow future high-throughput analyses to quickly assess the effects of specific drugs or blocking antibodies on EV uptake, which will facilitate the identification of the functional molecules involved in this process and the screening of potential treatments aimed at impairing EV uptake in pathological situations.

## Materials and Methods

### Antibodies

Primary antibodies employed were: anti-CD63 (Tea 3/10), and anti-CD9 (VJ1/20) mAbs (Immunostep)^36,37^ conditioned media from mouse hybridoma; polyclonal anti-EEA-1 (Santa Cruz) (1:50 for immunofluorescence) and polyclonal anti-GFP (Living colours, Clontech) (1:1000 for immunoblotting, 1:200 for immunofluorescence).

Secondary Abs employed were Goat-α-Mouse Alexa647 (Life technologies), Donkey-α-Goat Alexa647, Donkey-α-Rabbit Alexa488 and Phalloidin-Alexa647 (Invitrogen) (1:200 for immunofluorescence); Goat-α-Mouse HRP and Goat-α-Rabbit HRP (Thermo scientific) (1:5000 for immunoblotting).

### Cell culture

Breast cancer cell line SUM159 was cultured in DMEM F-12 culture medium supplemented with 5% FBS, 1% Penicillin/Streptomycin, Non-essential aminoacids (80 mg/ml)(HyClone GE Healthcare), HEPES (10 mM), Insulin (5 μg/ml) and Hydrocortisone (1 μg/ml). EV-depleted media was prepared by supplementing DMEM F-12 culture medium with FBS depleted from bovine EVs by ON ultracentrifugation at 120000 g. HEK293 cell line was cultured in DMEM culture medium supplemented with 10% FBS, 1% Penicillin/Streptomycin and HEPES (10 mM).

### Generation of CD9 and CD63-DSP constructs

The coding sequence of both human tetraspanins was amplified by PCR and subcloned in reading phase in the C-term end of the Dual Split Protein 1 (DSP1-7) construct^38^ kindly provided by Dr Zene Matsuda (Institute of Biophysics, Chinese Academy of Sciences). The full coding sequence of the fusion protein, as well as DSP1-7 (DSP1) and DSP8-11 (DSP2) were amplified again by PCR, sequenced and subcloned into pcDNA3 or PCR2.1 using the TOPO system (Invitrogen). PCR2.1 vector was digested with EcoR1 for subcloning of the DSP constructs into a lentiviral PLVX Puro vector for the generation of stably transduced cell lines.

### Cell transfection and transduction

SUM159 were transfected with DSP1/DSP2 constructs cloned into a pCDNA3 vector. Cells were trypsinized (1/2 P100 culture plate at 80% confluence), pelleted at 400 g and resuspended in 200 μL of incomplete DMEM F-12 medium supplemented with 5 μL of 1.5 M NaCl. A total of 20 μg of plasmid DNA was used per point of transfection. Electroporation conditions were 200 V, 975 μF in 4 mm width cuvette using a Biorad Gene PulserXCell^TM^. Cells were placed for 10–15 min on ice and then seeded in a p6 culture plate with complete culture medium.

For lentiviral production, HEK293 packaging cells were transfected with 5 μg of PLP1, 3.24 μg of PLP2, 2.16 μg of VSVG vectors and 2.76 μg of the plasmid encoding each of the DSP constructs in the PLVX vector, with lipotransfectine (Solmeglas) following manufacturer´s instructions. Culture supernatants obtained 48 h after transfection were incubated with SUM159 cells in the presence of polybrene (8 μg/mL)(Santa Cruz). Infected cells were selected with puromycin (1 μg/mL). In the case of single DSP transduction, infection efficiency was checked by transient co-transfection of the complementary construct in each case, and analysed by flow cytometry.

SUM159 cell lines that stably express DSP1-CD9/DSP2 or DSP1-CD63/DSP2 were selected by their green fluorescence signal at 488 nm by cell sorting. Cells transduced with DSP1-CD9 or DSP1-CD63 were labelled with VJ1/20 anti-CD9 or Tea3/10 anti-CD63 primary antibodies, respectively, and GαM Alexa 647 secondary antibody. Those cells with higher expression levels of the corresponding tetraspanin were sorted by its fluorescence at 647 nm using a BD Biosciences FACSAria Fusion cell sorter.

### EV enrichment

EVs were enriched from SUM159 conditioned media by ultracentrifugation. Secretory cells were cultured to confluence in p150 culture plates with 20 mL of DMEM F-12 supplemented with 5% EV depleted-FBS (centrifuged ON at 120000 g) for 5–7 days. Supernatants were collected and centrifuged 30 min at 4000 g to remove cellular remnants. Then supernatants were ultracentrifuged for 2 h at 100000 g to obtain an EV concentrate. Alternatively, in order to separate microvesicles and exosomes, supernatants were ultracentrifuged at 10000 g for 1 h to collect the resulting pellet enriched in microvesicles. The supernatant was ultracentrifuged again at 100000 g for 1 h to obtain the exosome-enriched pellet. Ultracentrifugations were performed in a Beckman L8-70M Ultracentrifuge using a SW-Bucket AH-627 rotor. Pellet obtained from a p150 culture plate was resuspended in 500 μl of 0.22 μm filtered PBS.

### BCA protein analysis

Protein concentration was assessed following the Pierce® BCA Protein assay kit protocol. Duplicates of sample’s absorbance at 540 nm were measured in a Tecan GENios Microplate reader.

### Nanoparticle tracking analysis

A 1/100 dilution of the sample diluted in filtered PBS was measured with a Nanosight NS500 equipment with the following parameters: camera level:10, Detection Threshold:10, 3 captures of 1 min. Data was analysed with NTA 3.1 Build 3.1.54 software.

### Western blot

Cells or EV samples were lysed with TBS + 1% Triton X-100 containing proteases inhibitors (Roche) at 4 °C for 30 min. Lysates were boiled in non-reducing Laemmli buffer at 96 °C for 5 min and 40 μl of lysates were loaded in 10% Polyacrylamide SDS-page gels.

After electrotransference with a Transfer-Blot Turbo system (BioRad), membranes were blocked with 5% skimmed-milk in TBS, 0.1% Tween-20 for 20 min. Immunoblots were revealed with Super Signal® West Femto HRP substrate (Thermo Scientific), and images acquired with a LAS 4000 mini system (General Electrics).

### Immunofluorescence microscopy

Coverslips were coated with 5 μg/ml fibronectin (Sigma-Aldrich) for 30 min at 37 °C, and 30000 cells/well were seeded in p24 well plates and cultured ON at 37 °C, 5%CO_2_. When indicated, EVs were added to the cells in a final volume of 400 μl of culture media and incubated for 5 h at 37 °C. Samples were washed with PBS and fixed with 4% paraformaldehyde for 10 min at RT. Coverslips were washed with TBS several times and where indicated, permeabilized by incubation with TBS, 1% Triton X-100 for 5 min, washed with TBS and blocked with TNB blocking solution (100 mM Tris-HCl, 150 mM NaCl, 0.5% blocking reagent PerkinElmer FP1020) for 30 min at RT. Samples were stained with primary antibodies ON in a wet chamber at 4 °C and appropriated secondary antibodies for 30 min at 37 °C and mounted with Fluoromount-G Aqueous Mounting medium (Sigma) containing 0.1 μM DAPI. Samples were analysed in an Axiovert200 (Zeiss) inverted microscope coupled to a CCD monochromatic camera (C9100-02 Hamamatsu) using 63X/1.4 oil Plan-Apochromat Ph3 objective. Excitation: SPECTRA-X (LUMENCOR) Illumination source DAPI 395/25; GFP 470/24; Cy5 640/30. Filters: GFP 503–538 nm (Semrock); Cy5 665LP (Chroma). Confocal microscopy images were acquired with a Leica TCS-SP5 confocal laser scanning unit attached to a Leica DMIRBE inverted epifluorescence microscope (Leica Microsystems, Heidelberg, Germany) using a HCX PL APO CS 100.0 × 1.40 OIL objective. Excitation: 405 nm laser for UV, He/Ne laser 488 nm for EGFP and Diode 633 nm laser for Alexa 647. Emission: for UV (422–477 nm), Green (494–563 nm), Far Red (726–800 nm). The effective pixel size was 0.3 microns/pixel. EGFP and Alexa647 signals were acquired simultaneously, but sequentially with UV signal.

### Renilla luciferase assay from lysates

Two p100 plates of SUM159 cells at 90% of confluence expressing DSP1 or DSP2 proteins were lysed with 500 μl of PLB (Passive Lysis Buffer from Dual-Glo® Luciferase Assay System, Promega) supplemented with Protease inhibitors (Roche) at 4 °C. EVs were recovered by ultracentrifugation from 40 mL of conditioned media from DSP1 or tetraspanin-DSP1 expressing cells and lysed in 500 μl PLB. 125 μl of DSP1-expressing EV- or cell-lysates were mixed with 75 μl of DSP2-expressing cell lysates and co-incubated for the indicated times in a p96 well clear bottom plate (Corning Incorporated® Costar®). Luminescence signal was obtained by addition of 50 μl of Renilla luciferase substrate Stop&Glo from the Dual-Glo® Luciferase Assay System and measured with a Tecan GENios Microplate reader at 5000 ms of exposure and a gain of 100.

### EV uptake luciferase assay

32000 target cells/well were cultured in a clear bottom p96 well plate for 24 h with EV-depleted culture media before being loaded with Enduren (Promega) by incubation for 2 h with 100 μl/well of a 60 μM Enduren solution in EV-depleted culture media. After the incubation, supernatant was removed. When indicated, receptor cells were fixed with 4% paraformaldehyde after Enduren loading and before EV addition. For the assessment of different chemical inhibitors, target cells preloaded with Enduren were pre-treated for 30 min with the indicated chemicals diluted in EV-depleted culture media in a total volume of 50 μl after Enduren supernatant removal.

Then 4–8 μg of EVs were added in a total volume of 100 μl of complete culture media. When inhibitors are used, EVs were added to target cells in the presence of the inhibitors. Before addition to cells, inhibitors from stock were diluted to the following indicated concentration of use^18^ in EV-depleted culture media. Chlorpromazine (7.5 μM) (CPZ); Nocodazole (10 μM) (NDZ), Cytochalasin D (1 μg/ml) (Cyt D), Genistein (200 μM) (Gen), Wortmannin (1 μM) (Wort) and Dynasore (Dyn) (80 μM) stocks prepared in DMSO; EIPA (75 μM) stock prepared in Methanol; Heparin (10 μg/ml) stock prepared in water. All of them from Sigma Aldrich.

When using the Dual-Glo® Luciferase Assay System, samples were lysed with PLB before the addition of Renilla luciferase substrate Stop&Glo.

All measurements were performed in duplicates and read at different times with a Tecan GENios Microplate reader, with 5000 ms of exposure time, gain 150.

To study how Enduren was processed and acquired by EVs, SUM159 were incubated for 2 or 6 h with 100 μl 60 μM Enduren diluted in EV-depleted culture medium. Supernatants from 6 wells of a p96 well plate were collected and pooled together. Cells were washed with PBS. Then 100 μl of PBS with Protease inhibitors were added per well. Cells were mechanically lifted and lysed by sonication with Sartorius LABSONIC M ultrasonic processor at 80% of amplitude, 0.6 cycles at 4 °C. Lysates were centrifuged at 21000 g to eliminate cellular remnants. 100 μl of either supernatants or lysates from cells pre-loaded with Enduren for 2 or 6 h were co-incubated in duplicates with 3 μg of DSP1-CD9/DSP2 EVs and luminescence signal was measured at different time points with a Tecan GENios Microplate reader, with 5000 ms of exposure time, gain 150.

### Annexin V-PE/7AAD viability protocol

Cells were cultured in p24 well culture plates and treated with the different inhibitors at the same concentrations previously indicated. Both supernatants and cells lifted with trypsin were collected, washed twice with cold PBS and resuspended in annexin binding buffer (50 mM HEPES, 700 mM NaCl, 12.5 mM CaCl2, pH 7.4) at a concentration of 1 × 10^6^ cells/mL. 100 μl of this solution of cells were stained for 15 min at RT with 2.5 μl of AnnexinV-PE and 5 μl of 7AAD (BD Pharmigen^TM^ 559763). Cells were analysed by flow cytometry using a Becton Dickinson FACSCalibur flow cytometer. PE detection: Emission wavelength at 488 nm and detection at 585/42 nm, 7AAD detection: Emission wavelength at 488 nm and detection at 660/13 nm. Results were analysed with FlowJoV-10 program.

### Statistical analyses

Data analysis was performed using GraphPad Prism version 6.0 (GraphPad Software Inc, CA, USA). Interaction, F-values and degrees of freedom from the ANOVA analyses of all graphs are included in the Supplementary Table.

## Results

### EV-DSP constructs development and characterization

Our main objective is to develop a quantitative and highly sensitive method to measure EV uptake, enabling us to explore the underlying molecular mechanisms controlling this process. The proposed strategy is based on the use of two reporter proteins, DSP1 and DSP2, which work as tags encoding non-functional split versions of EGFP and Renilla luciferase proteins. When present in the same cellular compartment, DSP1 and DSP2 can associate to give rise to a complete protein displaying fully recovered green fluorescence and luciferase activity. With the aim of targeting DSP1 reporter protein specifically to extracellular vesicles (EVs) we designed chimeric proteins encompassing DSP1 fused to either CD9 or CD63 human tetraspanins (Supplementary Fig. S1), which are widely used as EV markers. Since the C-terminal sequence of tetraspanins has been reported to be functionally active by engaging in interactions with an array of different intracellular proteins^39^, we decided to fuse the DSP1 tag to the N-terminus of the tetraspanin protein. The four different constructs (DSP1-CD9, DSP1-CD63, DSP1 and DSP2) were thereafter subcloned into both the pCDNA3-TOPO vector, allowing for transfection and transient expression in eukaryotic cells, and into the pLVX-Puro lentivirus vector for viral transduction and stable cellular expression (Supplementary Fig. S1).

**Figure 1:**
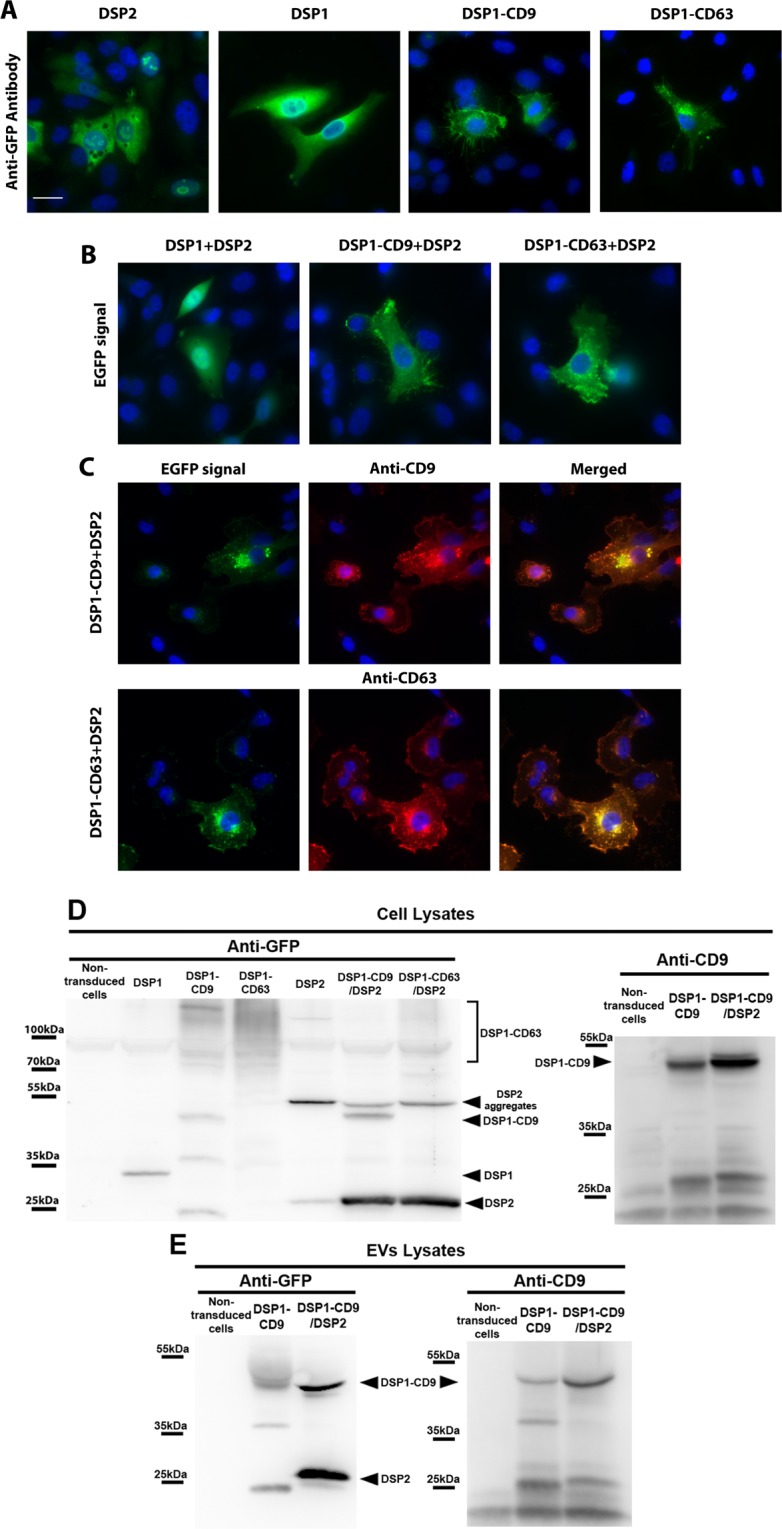
Characterization of DSP constructs. (**A**) Subcellular localization of the different DSP constructs. SUM159 cells were transiently transfected with the indicated DSP constructs and stained with α-GFP rabbit polyclonal Ab. **(B)** Dual Split Proteins reassociation detected by fluorescence microscopy. SUM159 cells were co-transfected with DSP1 + DSP2, DSP1-CD9 + DSP2 or DSP1-CD63 + DSP2 and EGFP signal was observed by fluorescence microscopy 48 h after transfection. **(C)** Staining of DSP contructs with anti-tetraspanin antibodies. SUM159 cells were co-transfected with DSP1-CD9 + DSP2 or DSP1-CD63 + DSP2 and stained with VJ1/20 anti-CD9 or Tea3/10 anti-CD63 mAbs, respectively. EGFP signal and anti-tetraspanin stainings were observed by fluorescence microscopy 48 h after transfection. All samples were co-stained with DAPI. Images were acquired in a wide-field epifluorescence microscope. Bars = 20 μm. **(D)** SUM159 cell lines that stably express each DSP construct were obtained by transduction with a pLVX lentiviral vector, lysed and subjected to SDS-PAGE. **(E)** Lysates from EVs isolated by ultracentrifugation at 100000 g from SUM159 expressing DSP1-CD9 or DSP1-CD9/DSP2 conditioned media. Membranes were blotted with α-GFP rabbit polyclonal antibody or α-CD9 (VJ1/20) mouse mAb. Positions of molecular weight markers and the different specific bands are indicated.

The subcellular localization of the DSP constructs was assessed by fluorescence microscopy in SUM159 human breast cancer cells. When transiently transfected cells were stained with an anti-GFP antibody, both DSP1 and DSP2 proteins were found to be diffusely localized in the cytoplasm and nucleus, while DSP1-tetraspanin constructs (DSP1-CD9 or DSP1-CD63) showed a similar subcellular localization to that characteristic of the endogenous tetraspanins, being both tagged tetraspanins localized at the plasma membrane and vesicle compartments although DSP1-CD63 was more restricted to intracellular vesicular compartments (Fig. 1A) as usually described. When DSP1, DSP1-CD9 or DSP1-CD63- expressing cells were transiently co-transfected with DSP2, DSP tags were able to reassociate, with concomitant recovery of the green fluorescence. Localization of this green fluorescence signal also paralleled the patterns of expression of soluble GFP, CD9 or CD63 (Fig. 1B). Subcellular localization and immunoreactivity of the fusion proteins was also corroborated by staining with the corresponding specific anti-tetraspanin antibodies (Fig. 1C).

In order to generate a high-throughput system, SUM159 cells were transduced with lentiviral vectors for stable expression of each DSP construct (DSP1, DSP1-CD9, DSP1-CD63 or DSP2) or double combinations (DSP1/DSP2; DSP1-CD9/DSP2, DSP1-CD63/DSP2). The cellular expression of the DSP-tagged proteins and their sorting into EVs was assessed by western blot. In cell lysates, DSP1 was resolved as a band migrating just under 35 kDa while DSP2 was detected as two bands of ≈25 kDa and ≈55 kDa. DSP1-CD63 appears with a smear pattern around 110 kDa because of the different glycosylated forms of CD63 that coexist in the cell. DSP1-CD9 was detected both in cellular and EV lysates as a band of ≈50 kDa, while endogenous CD9 was detected as a double band migrating at around 25 kDa (Fig. 1D,E). A very high expression of exogenous proteins was achieved in double transduced cells, compared to single transduction.

**Figure 2:**
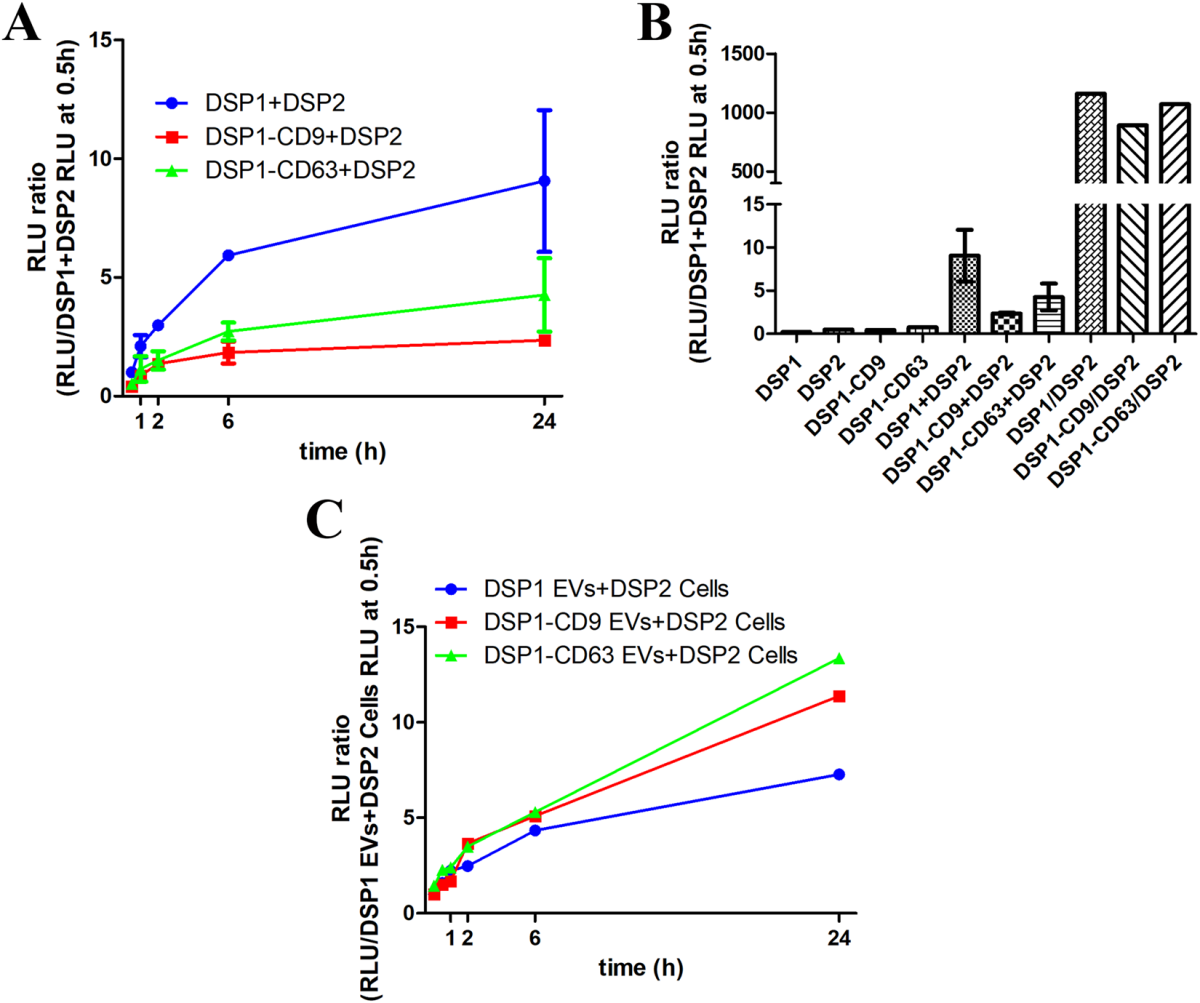
DSP1 and DSP2 *in vitro* re-association assessment. (**A)** DSP1 and DSP2 reassociation kinetics in cell lysates. SUM159 stably expressing DSP1, DSP1-CD9 or DSP1-CD63 were lysed and each lysate was mixed with a cell lysate from DSP2-expressing cells. Renilla luciferase activity was measured with Stop&Glo Renilla reagent at the indicated times. n = 2. (**B**) Comparison of the signal obtained with Stop&Glo reagent at 24 h from combinations of cell lysates of cell expressing DSP1 constructs with cell lysates from cells expressing DSP2 (DSP1 + DSP2, DSP1-CD9 + DSP2 and DSP1-CD63 + DSP2) and with cell lysates from cells that co-expressed both constructs (DSP1/DSP2, DSP1-CD9/DSP2 and DSP1-CD63/DSP2). Negative controls of independent lysates from cells expressing either DSP1, DSP2, DSP1-CD9 or DSP1-CD63 are depicted. **(C)** Reassociation kinetics with EV lysates. EVs were isolated from conditioned media of SUM159 stably expressing DSP1, DSP1-CD9 or DSP1-CD63 by ultracentrifugation at 100000 g, lysed and each lysate was mixed with a cell lysate from DSP2-expressing cells. Renilla luciferase activity was measured with Stop&GloRenilla reagent at the indicated times.

To assess the recovery of Renilla luciferase activity upon DSP association, cell lysates from cells expressing DSP1, DSP1-CD9 or DSP1-CD63 were mixed in a 1:1 ratio with cell lysates from DSP2-expressing cells (Fig. 2A). A higher luciferase signal was observed when incubating DSP2 lysates with DSP1-containing lysates than with DSP1-tetraspanin fusion proteins, although the kinetics of association were quite similar, reaching almost the maximum signal at 6 h (Fig. 2A). The highest luciferase signal was detected by directly using lysates from double-transduced cells, while no signal was detectable in lysates from single-transduced cells alone (Fig. 2B). In parallel assays, lysates of EV released by cells transduced with the different DSP1 constructs (DSP1, DSP1-CD9, DSP1-CD63) were incubated with cellular lysates from DSP2-transduced cells (Fig. 2C). In these experiments, a stronger signal at 24 h was achieved with the EV containing the DSP1-tetraspanin constructs than with EVs obtained from cells expressing DSP1 alone, probably as a result of the relative enrichment in these constructs caused by tetraspanin sorting to EVs. However, the signals obtained were rather scant and only detectable at long times of incubation (Fig. 2C).

### EV uptake detection

As a next step EV uptake assays were performed by incubating DSP1-CD9 containing EVs with cells expressing DSP2. In these experiments we employed two different Renilla substrates to detect luminiscence output signal, the classical Stop&Glo™ reagent, which requires lysis of the samples for measurement, and Enduren™, a cytopermeable substrate that does not require cell lysis and thus, would allow kinetic assessment of EV uptake by the cells. However, we could not detect any luminiscence signal at either 6 or 24 h with either of the two substrates, indicating that no DSP1-DSP2 reassociation had occurred in our samples.

**Figure 3:**
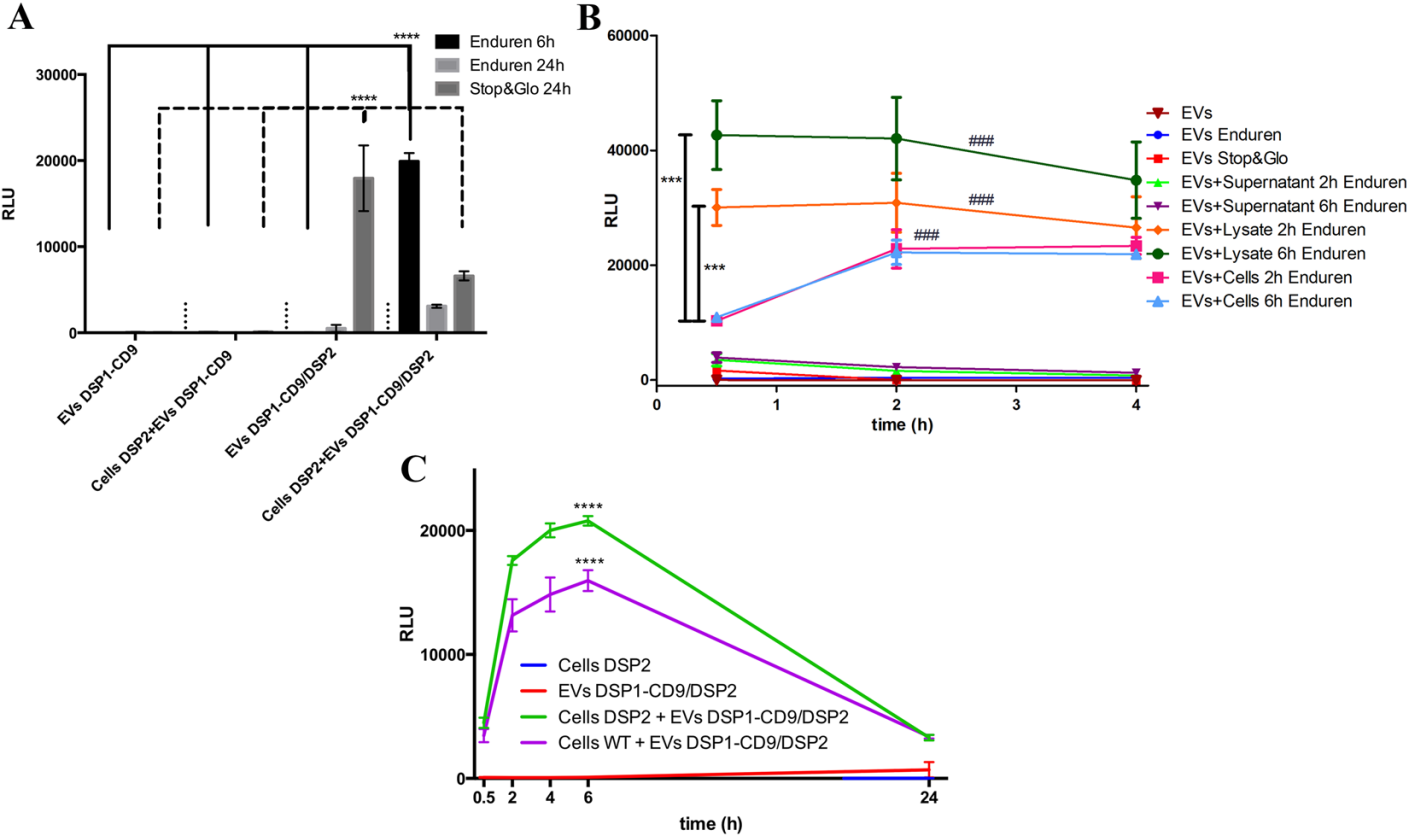
EVs uptake assay. (**A)** DSP2-expressing target cells were preloaded with Enduren and incubated with DSP1-CD9 or DSP1-CD9/DSP2 EVs to be measured either at 6 or 24 h. Alternatively, DSP2-expressing target cells were incubated with EVs for 24 h, lysed and measured after Stop&Glo Renilla luciferase substrate addition. Signal of isolated DSP1-CD9 or DSP1-CD9/DSP2 EVs is also shown. Statistics: Statistics: ****. Two way Anova, Tukey’s multiple comparison test. n = 4. **(B)** DSP1-CD9/DSP2 EVs were incubated with supernatants or with mechanical obtained lysates from SUM159 WT cells preloaded with Enduren during 2 or 6 hours. EVs also were added to alive cells preloaded with Enduren at 2 or 6 hours. Controls of EVs incubated with Enduren or Stop&Glo reagent without EVs lysis are also shown. Luminescence signal from EVs with the different reagents and conditions at shown time points of incubation are depicted. Statistics: *** comparisons between EVs in the presence of cell lysates or alive cells at 0.5 hours; ### compared to EVs in the presence of cell supernatants and controls. Two way Anova, Tukey’s multiple comparison test. n = 3 **(C)** EV uptake kinetics using DSP2 or untransduced (WT) as target cells, pre-loaded with Enduren and incubated with DSP1-CD9/DSP2 EVs. Signal of either cells or EVs alone are also depicted. Statistics: **** compared to negative controls. Two way Anova, Tukey’s multiple comparison test. n = 4.

As a positive control, we included in the measurement EVs isolated from the conditioned media of cells stably co-transduced with both DSP1-CD9 and DSP2, that would carry fully competent intraluminal Renilla. To our surprise, EVs expressing both DSP1-CD9 and DSP2 provided a clear luminiscence signal only with the classical Stop&Glo substrate, after lysis of the EVs, but we observed no signal using the Enduren substrate with intact EVs. However, as soon as those EVs were placed in contact with Enduren-loaded target cells, luciferase signal was readily detected (Fig. 3A).

Enduren substrate has the advantage of being permeable through the plasma membrane, but cannot be directly processed by Renilla enzyme, since it requires a previous step of deprotection by cellular esterases to produce the active Renilla substrate Coelenterazine-h. The fact that isolated EVs carrying fully compentent Renilla did not provide any signal when incubated with Enduren could be due to a differential permeability of Enduren to EV membranes or to the lack of esterase activity in EVs, necessary to deprotect Enduren. To investigate the behaviour of Enduren signal on EVs we performed an additional experiment in which EVs isolated from DSP1-CD9/DSP2 expressing cells were directly incubated with Enduren or the substrate from the Stop&Glo kit (without previous lysis). Again, no signal could be observed at any time point (Fig. 3B). Then, EVs were exposed to cell lysates (detergent-free mechanically obtained by scrapping and sonication) from cells that had been loaded with Enduren and incubated with this substrate for 2 or 6 h before lysis. Under these conditions, a robust signal could be observed. A higher signal was achieved when incubating EVs with cell lysates from cells that had been loaded with substrate for 6 h, than with lysates of cells loaded with Enduren for 2 h before lysis, suggesting that Enduren deprotection is a continuous process in the cell. However, the signal remained thereafter stable at the different measurement times, indicating that substrate availability is the main factor determining the amplitude of the signal. These results would indicate that cellular esterases are able to deprotect Enduren to produce the active substrate Coelenterazine-h, which can readily traverse EV membranes. In contrast, when EVs were incubated with conditioned media from cells loaded with Enduren (for 2 h or 6 h prior supernatant collection) no signal could be observed, indicating that either coelenterazine does not escape from the cell, or that its low stability in solution prevents signal detection. Finally, when EVs carrying Renilla enzyme (DSP1-CD9/DSP2) were added to live Enduren-loaded cells, a dynamically increasing signal could be observed, indicating a gradual exposure of Renilla to the active substrate. In this case, no quantitative differences could be detected between those samples in which EVs were added to cells that had been loaded with Enduren for 2 or 6 h prior to EV addition, suggesting that the limiting factor for the signal in this case is not the amounts of processed Enduren, but its availability to the enzyme that is only possible upon the gradual uptake of EVs by the target cells (Fig. 3B).

These results suggested that a complete cellular machinery was needed for Enduren deprotection and prompted us to define a new strategy for measurement of EV uptake. This new assay is based on the intrinsic luciferase activity of EVs derived from DSP1-CD9/DSP2 expressing cells, rather than on DSP reassociation. To measure EV uptake with this new approach, target cells are preloaded with the cytopermeable Renilla luciferase substrate Enduren, and after washing the excess of reagent, EVs isolated from conditioned media of DSP1-CD9/DSP2 double-transduced cells are added. The detected signal will come only from the fraction of EVs being uptaken, which allows Renilla luciferase to gain access to the deprotected coelenterazine substrate available inside the target cells (Fig. 7). With this new strategy in mind, we performed a full kinetic analysis (Fig. 3C). Again, no signal was detected at any time point in intact EV samples incubated with Enduren (Fig. 3C red line). Only when EVs were uptaken by target cells, the substrate was accessible to the Renilla luciferase (Fig. 3C green line), giving measurable signal as early as after 30 min of incubation. With this alternative approach, the luminescence signal is not dependant on DSP2 expression on target cells, and a positive signal can be observed with wild-type untransfected target cells (Fig. 3C, purple line) so in all subsequent experiments wt recipient cells were used.

**Figure 4:**
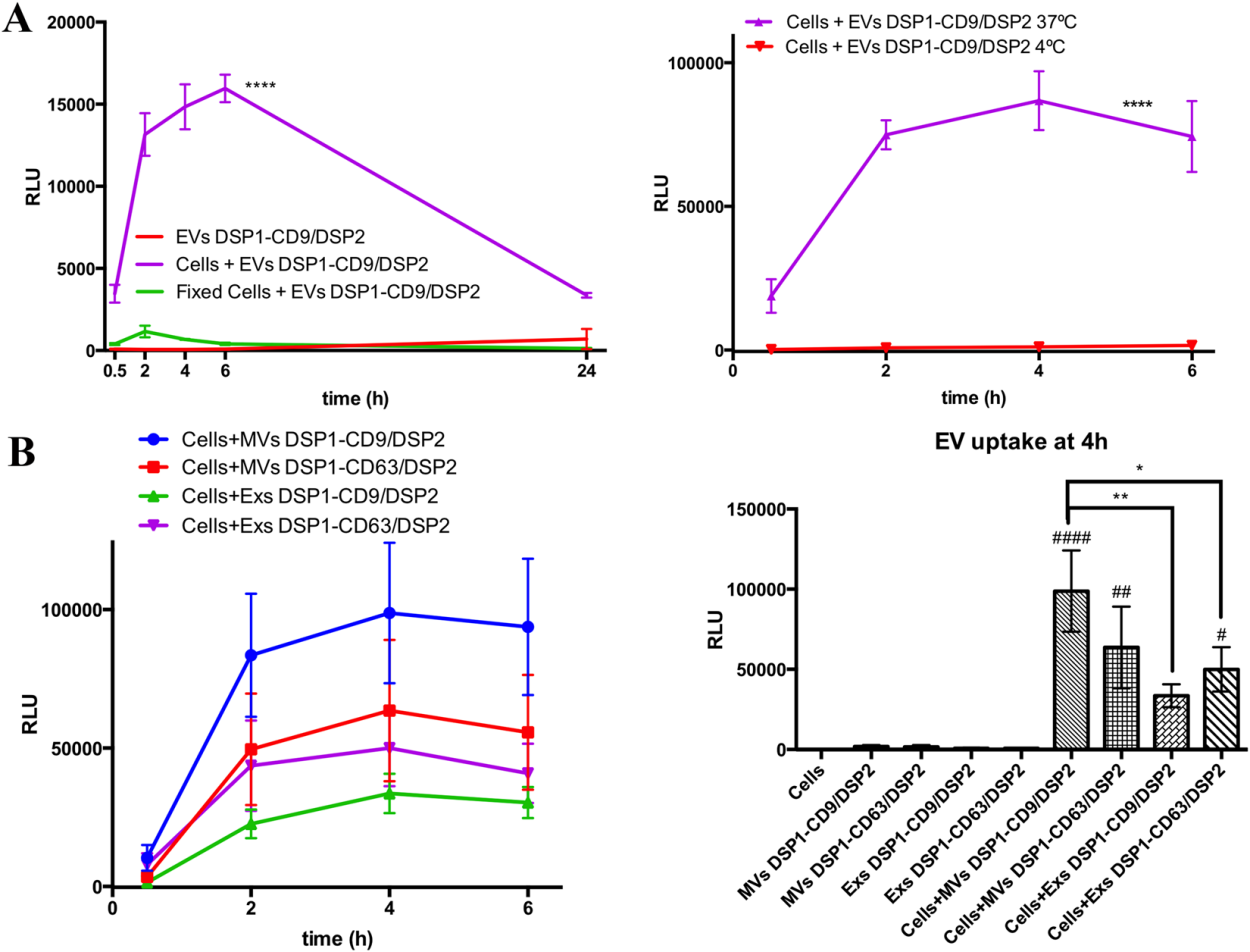
EV uptake assay characterization. (**A)** EV uptake assays performed with receptor cells preloaded with Enduren and either fixed with 4% PFA (left plot) or performed at 4 °C (right plot). Statistics: **** comparison between non-fixed (purple) and fixed cells (green). Two way Anova, Tukey’s multiple comparison test. n = 4. EV uptake assay performed at 37 °C or at 4 °C (right plot). Statistics: **** compared to positive control at 37 °C (purple). Two way Anova, Dunnett’s multiple comparison test, n = 3. **(B)** EV uptake assay with EVs obtained by differential ultracentrifugation. Microvesicles (MVs) or Exosomes (Exs) from DSP1-CD9/DSP2 or DSP1-CD63/DSP2 cells were used. EV uptake kinetics are represented in the left plot. Bar plot of EV uptake signal at 4 h including all negative controls is shown in the right plot. 5.58 × 10^9^ EVs of each type were used per well. Statistics: **,* comparison between EV types; ####,##,# comparison with all the controls; p values = 0.0034**, 0.0395*, < 0.0001####, 0.0019##, 0.0311#. Two way Anova, Tukey’s multiple comparison test. n = 4.

A series of additional control experiments were performed in which we could observe that fixation of the target cells after 2 h of Enduren loading and prior to EV addition (Fig. 4A, left plot) or incubation of EVs with target cells at 4 °C (Fig. 4A, right plot) impaired the luciferase activity, suggesting that we are measuring a process that requires an active cellular metabolism.

So far, all experiments have been performed with a total EV preparation obtained by direct ultracentrifugation at 100,000 g, of conditioned media from EV producing cells, where both exosomes and microvesicles are pelleted. For further characterization of the system, we performed differential sequential ultracentrifugations to enrich the samples in large EVs, mostly microvesicles (MVs), and small EVs, mainly exosomes (Exs), from the conditioned media of cells that either express DSP1-CD9/DSP2 or DSP1-CD63/DSP2. In these analyses the same number of vesicles (as determined by NTA) was added to the wells containing the recipient cells. Consistent with their bigger size and therefore higher molecular content, the highest signals were obtained with larger EVs. However, when comparing DSP1-CD9/DSP2 with DSP1-CD63/DSP2, the first gave bigger signals with MVs, while the latter provided a better signal with exosomes, consistent with the different subcellular distribution of both tetraspanins (Fig. 4B).

### Proof of concept of the quantitative EV uptake assay

**Figure 5:**
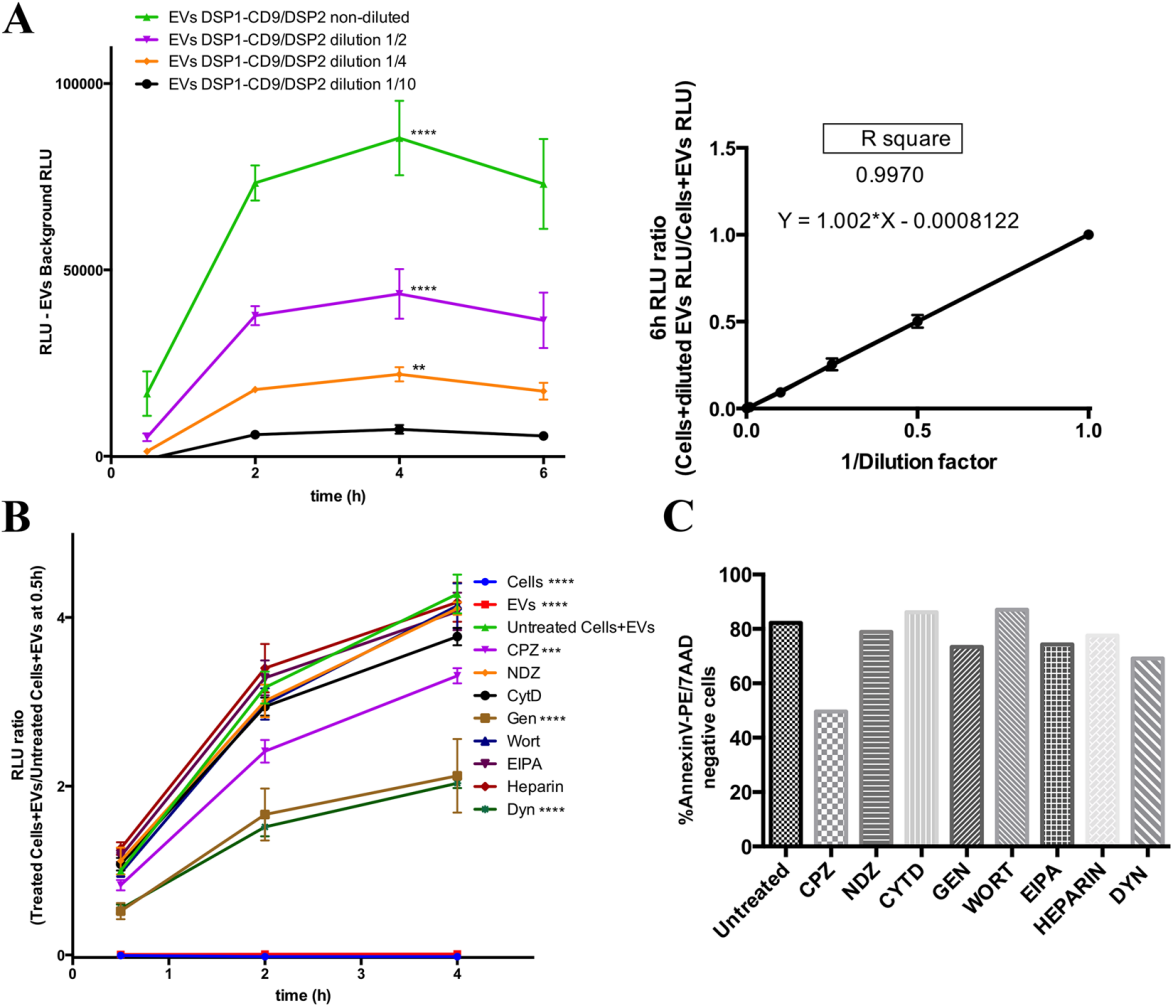
Proof of concept of the quantitative assay. (**A)** Dose-response EV uptake assay. SUM159 target cells were incubated with several dilutions of DSP1-CD9/DSP2 EVs. 3.8 × 10^10^ EVs per well were used in non-diluted samples. Statistics: ****, ** compared to negative control of DSP1-CD9/DSP2 EVs alone at 4 h; **** p < 0.0001; **p < 0.001. Two way Anova, Tukey’s multiple comparison test, n = 3. Linear regression between the dilution factor of EVs and the RLU ratio (Cells + EVs RLU/Cells + diluted EVs) is shown in the right graph. **(B)** Non-transduced SUM159 target cells were loaded with Enduren, and then pretreated for 30 min with different chemical inhibitors^18^: Chlorpromazine (7.5 μM) (CPZ); Nocodazole (10 μM) (NDZ), Cytochalasin D (1 μg/ml) (Cyt D), Genistein (200 μM) (Gen), Wortmannin (1 μM) (Wort), EIPA (75 μM), Heparin (10 μg/ml) and Dynasore (Dyn) (80 μM). Then DSP1-CD9/DSP2 EVs isolated by ultracentrifugation were added, in the presence of the inhibitors. Data were normalized in each measurement time point to untreated Cells + EVs at 0.5 h. Statistics: ****, *** differences with positive control (untreated Cells + EVs); p values = < 0.0001****, 0.0002***. Two way Anova, Tukey’s multiple comparison test. n = 4. **(C)** Annexin/7AAD Cell Viability assay. The percentage of double negative cells for Annexin and 7AAD stainings was measured by flow cytometry after 4 h of treatment with each chemical inhibitor.

To assess the quantitativeness of the system we analysed the dose-dependence of the signal (Fig. 5A). When diluting EV samples, luciferase signal decreases almost in the same proportion as the dilution factor (1:2, 1:4, 1:10 or 1:100), resulting in a proportional relation that nicely fits into a linear regression (Fig. 5A, right plot). Signal obtained with 1:100 dilution (corresponding to 3.8 × 10^8^ EVs/sample) was no longer statistically significantly different to EV background (data not shown). If we consider that the limit of detection of the measurement would be the signal of EVs alone ± 3 times the standard deviation of their measure, the detection limit of our measure would correspond to 9.35 ± 2.46 × 10^8^ EVs/well.

As a proof of concept of the use of the assay as high-throughput screen platform, receptor cells were treated with several drugs that inhibit or impair different uptake pathways (Fig. 5B). Drugs used were chlorpromazine, a clathrin inhibitor; nocodazole and cytochalasin D, that respectively block tubulin or actin dynamics; genistein, a general Tyr kinase inhibitor that indirectly inhibits dynamin2; wortmannin, a PI3K inhibitor that inhibits macropinocytosis; EIPA, a Na^+^/H^+^ exchanger inhibitor that also inhibits macropinocytosis; heparin, which was used as a lectin competitor; and dynasore, which is a dynamin2 inhibitor. In these analyses using SUM159 as target cells, the highest reduction in EV uptake was observed by inhibiting dynamin2 function either directly with dynasore, or indirectly by genistein. A milder reduction was also produced by chlorpromazine (Fig. 5B). The viability of cells treated with these different drugs was assessed in parallel to rule out any possible toxic effects of these treatments (Fig. 5C), and only a moderate toxicity was detected with chlorpromazine treatment, at the doses and times employed in the assays.

### EV uptake detection by confocal fluorescence microscopy

**Figure 6:**
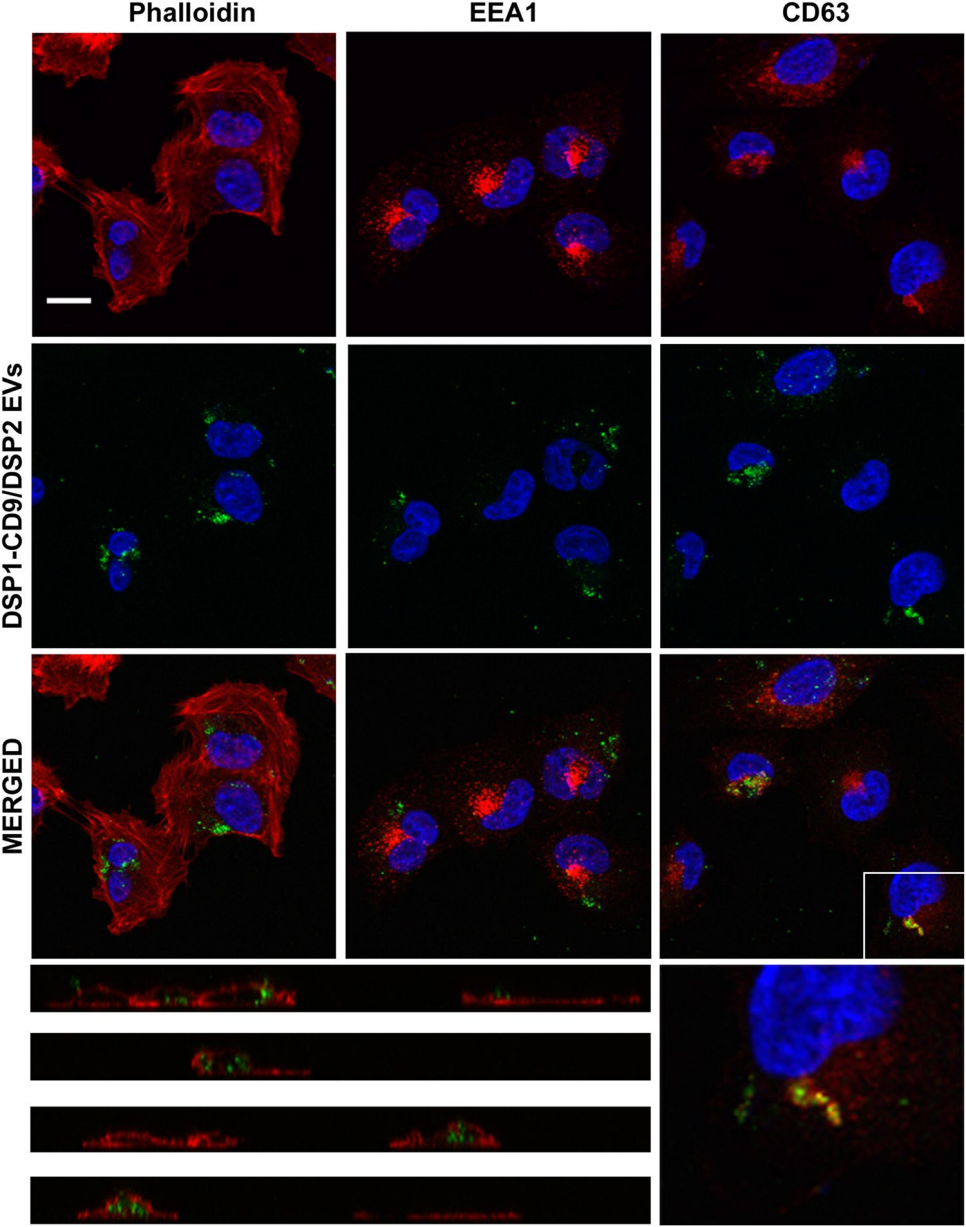
DSP1-CD9/DSP2 EVs uptake detected by fluorescence confocal microscopy. SUM159 target cells were incubated with DSP1-CD9/DSP2 EVs isolated by ultracentrifugation. Cells were fixed and permebilized after 5 h of incubation with EVs. Samples were stained with Phalloidin-647, αEEA1 antibody or αCD63 (Tea 3/10) mAb. All samples were co-stained with DAPI and analysed in a confocal fluorescence microscope. A maximal projection of the central optical sections is shown. Below, a series of vertical sections of Phalloidin-stained samples are depicted, clearly showing the intracellular localization of uptaken EVs. On the right, a zoomed image in which colocalization of EVs with CD63 labelling is demonstrated on a single confocal plane. Bar = 10 μm.

A major advantage of DSP constructs is that they possess both EGFP (green fluorescence) and Renilla (luminescence) activities, allowing for multiple ways of detection. Therefore, DSP1-CD9/DSP2-containing EVs were also used to follow the subcellular localization of uptaken EVs using confocal fluorescence microscopy. At 5 h of incubation most cells showed an accumulation of GFP signal that could lie more or less close to the nucleus. However, some cells sowed a more dispersed dotted pattern. The immunolabeling methods for the three conditions were the same and performed at same time. In these experiments, we were able to clearly detect partial colocalization of uptaken DSP1-CD9/DSP2 EVs with endogenous CD63 (Fig. 6, right panels and zoomed image) inside some of the target cells (Fig. 6, bottom vertical sections), but not with EEA-1 or the actin cytoskeleton (Fig. 6).

In sum, the proposed method combines the feasibility of exploring subcellular localization of uptaken EVs with the high sensitivity of luminescence measurements. By using a cytopermeable Renilla substrate, we could exclusively detect the signal from uptaken EVs. This method was shown to be highly sensitive and quantitative, allowing a dynamic follow-up in a high-throughput scheme to unravel the molecular mechanisms of EV uptake in different biological systems.

## Discussion

Since EV size lies under the resolution limit of most optical techniques, several artefacts can occur when measuring their binding or uptake by target cells. When fluorescently-labelled EVs are used, discrimination between EV binding or uptake may be misleading, and in some cases, treatment of target cells with trypsin has been reported to greatly reduce the signal^40^. Additional artefacts may arise by the use of lipophilic dyes, which even after washing the non-incorporated excess, can be exchanged from the EV membrane into that of the target cell without involving a real active uptake process. Thus, we decided to generate a system that would allow us to discriminate between binding and uptake of EVs by target cells, and that could be easily adapted to a high-throughput screening format.

The DSP reporter proteins have already been used to measure viral entry^34^ as well as the uptake of exosomes produced by the flagellate Trichomonas protozoa^41^. In order to translate this system to mammalian cells, we decided to fuse DSP1 to the N-terminal sequence of the human tetraspanins CD9 and CD63 (Supplementary Fig. S1), that are ubiquitously expressed by most cell types and preferentially sorted into EVs, being amongst the most abundant transmembrane proteins on their surface^42^.

We confirmed the correct subcellular localization and incorporation of the fusion proteins in EVs by staining with anti-GFP antibodies, and confirmed that the DSP1-tetraspanin and DSP2 constructs are able to reassociate when expressed in the same cell (Fig. 1). Western blot analyses revealed that a greater overexpression was achieved when DSP1-CD9 was co-expressed in the producing cells with DSP2 (Fig. 1D,E). This effect could be due to the sorting strategy employed to generate the stably-transduced cells (since it is easier to sort GFP-positive cells, while DSP1-CD9 transduced cells were selected by means of CD9 overexpression on their surface over a background of endogenously expressed CD9), or alternatively, because reassembly of the DSP1 fusion proteins with DSP2 in the producing cell during early protein biogenesis may enhance their stability and facilitate higher overexpression. Regarding DSP2, two different bands were observed in western-blot analyses of cell lysates, and some patchy appearance in the nucleus was detected by fluorescence microscopy. The upper band of DSP2 could not be observed in lysates of EVs, suggesting that DSP2 may exist both as a soluble protein in the cytoplasm and also forming aggregates, which are excluded from EVs (Fig. 1D,E). These aggregates are commonly seen in proteins conformed by non-paired β-sheets as it is the case for DSP2, which encompasses the 8–11 β-sheets from GFP^33,43^.

The initial method envisioned to quantitate EV uptake, relied on the association of DSP1-tetraspanins from EVs with DSP2 expressed in soluble form in the target cells. Since DSP tags are split molecules that are not individually endowed with reporter activity, their association is an absolute requirement for activation of the two reporter signals: green fluorescence and luminescence. We were able to confirm that the fusion proteins are indeed associated when co-expressed in the same cell and when mixing cell lysates from single transduced cells. In these *in vitro* conditions with cell extracts, we observed that the association kinetics reached its maximum at 6 h, being the luminescence signal much higher when lysates from DSP1 expressing cells were allowed to associate with DSP2 than when lysates from DSP1-tetraspanin (either DSP1-CD9 or DSP1-CD63) expressing cells were mixed with DSP2 containing lysates. Moreover, cellular lysates from cells expressing both constructs DSP1-CD9 and DSP2; DSP1-CD63 and DSP2 or DSP1 and DSP2 displayed a three-fold higher signal than any other condition tested (Fig. 2B). This difference may be due to a more efficient association during the simultaneous translation of both proteins, or to the higher expression achieved in double transduced cells. When EV lysates were incubated with cell lysates containing DSP2, a stronger signal was achieved with DSP1-tetraspanin containing EVs, suggesting a better incorporation and selective enrichment of these fusion proteins into EVs over DSP1 alone (Fig. 2C), however, the signal obtained with EV lysates was dim and displayed a very slow and delayed kinetics. When the designed EV uptake luciferase assay was performed by incubating DSP2 receptor cells with DSP1-CD9 containing EVs, no luminescence signal could be detected (Fig. 3A). These results would indicate that no reassociation of the two tags is attained even after 24 h of incubation, suggesting that no direct fusion of EV membrane with the target cell plasma membrane is taking place, and that cytoplasmic DSP2 cannot readily access the endocytic vesicles putatively containing the internalized EVs with luminal DSP1. In agreement with the lack of direct fusion in our cellular system, analysis by confocal fluorescence microscopy shows that some uptaken EVs colocalized with CD63 inside the receptor cell (Fig. 6), indicating that they could be eventually incorporated into endosomal compartments^44^.

**Figure 7:**
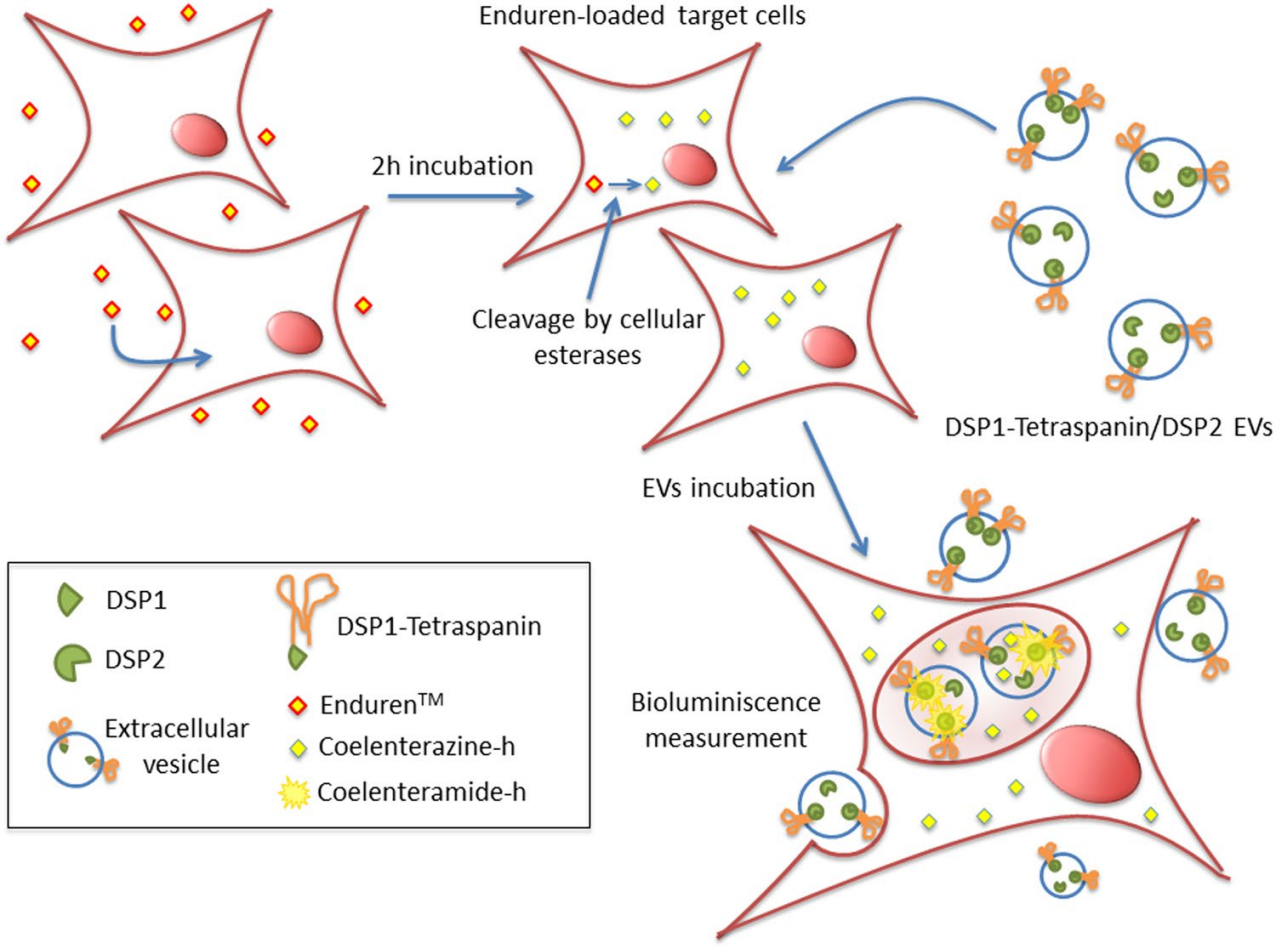
Scheme of the EV uptake assay. First, receptor cells are preloaded with Enduren, a cytopermeable protected Renilla luciferase substrate. Once internalized, Enduren is processed by intracellular esterases, rendering Coelenterazine-h Renilla luciferase substrate. EVs are isolated from cells that stably-express both DSP2 and either DSP1-CD9 or DSP1-CD63, and thus carrying fully competent Renilla enzyme. Only those EVs that are taken up by target cells and internalized will gain access to the deprotected Renilla luciferase substrate and will transform it into Coelenteramide-h, which emits the luminescence signal.

Thus, a new strategy was designed based on the observation of the behaviour of the luminescence signal obtained with the cytopermeable protected Renilla luciferase substrate, Enduren™. This substrate can be readily taken up by cells, and after traversing the plasma membrane is deprotected and converted to the active Coelenterazine-h substrate by esterase cleavage. Intriguingly, with isolated EVs obtained from conditioned media of cells expressing both DSP1-CD9 and DSP2, that displayed green fluorescence, we could not detect signal with Enduren reagent, but yielded a strong signal with the classical Stop&Glo™ substrate after their lysis (Fig. 3A). Thus, DSP1-CD9/DSP2 containing EVs did indeed possess Renilla activity but either they could not incorporate Enduren, or they did not contain esterase activity to convert it to the active Coelenterazine-h form. To directly address this issue, we incubated those EVs with lysates from mechanically lysed cells that had been preloaded with Enduren, and then a clear signal could be observed (indicating that the converted Enduren substrate can indeed access the Renilla enzyme inside EVs). We also included in the assay conditioned media from Enduren-loaded cells after 2 or 6 hours of loading, to ensure that the converted Enduren is not released from the cells. No signal could be detected by incubating EVs with those supernatants (Fig. 3B). These essential controls support the idea that EVs either lack esterases, so that Enduren cannot be processed by isolated EVs, or that EV membrane is impermeable to unprocessed Enduren but permeable to the processed form. In any case, these controls clearly demonstrate that Renilla signal is only observable in our method after active uptake by Enduren-loaded cells, that provide substrate accessibility to the enzyme. Taking this into account we propose a modified version of the assay, which is based on measuring the uptake of EVs carrying both DSP1-CD9/DSP2 constructs by receptor cells that have been preloaded with the Enduren substrate (Fig. 7).

This modified assay fulfils the desired requirements for the measurement since it can discriminate between EV binding (that could be detected by their EGFP fluorescence or by their luminescence with classical Renilla substrates after lysis) and uptake (luminescence with the Renilla substrate Enduren). In addition, it presents a series of advantages over the first approach: (i) because the EV producing cells are co-transfected with both DSP1 (DSP1, DSP1-CD9 or DSP1-CD63) and DSP2 constructs, they show EGFP fluorescence, and can be easily sorted for stable transfection, allowing for easy selection of highly expressing cell sublines; (ii) since target cells are only required to provide enough substrate supply and accessibility, any type of cell could be employed as target cells without the need to be transfected, thus greatly improving the flexibility of the assay; (iii) since DSP1-DSP2 association takes place during early protein biosynthesis, there is no lag time due to DSP association during the measurement, improving kinetic analyses at initial times of the assays; and (iv) since no cell lysis is required for the measurement, the same samples in a single 96-well plate can be followed over different time points. In these time-course analyses a maximal signal could be observed at 4–6 h of incubation of target cells with EVs, with a decline at 24 h (Fig. 3C). This decline could also be due to substrate exhaustion, so analyses at longer times may require additional Enduren supply.

The good sensitivity and linearity of the assay was proven in a dose-response experiment, which revealed that the limit of detection may be around 9.5 10^8^ EVs/well (Fig. 5A). Of course, we can only quantitate the number of particles we add to the experiment, but not the actual number being uptaken by the cells, which should be only a portion of the added vesicles. This dose-dependence was also observed when evaluating the signal obtained by uptake of large vesicles versus exosomes (Fig. 4B). Taking into account that the assay was performed with the same number of vesicles per condition, the highest signal seen with MVs may directly relate to their bigger size compared with exosomes, being able to accommodate on their surface (that increases with the square of the radius) significantly more DSP1-CD9/DSP2 or DSP1-CD63/DSP2 molecules. These analyses also revealed the different segregation of tetraspanins, being DSP1-CD9 more abundant in larger vesicles because of the high expression of CD9 on the plasma membrane, than CD63, which is especially enriched in smaller size vesicles that derive preferentially from the endosomal compartment^42^. However, differential ultracentrifugation is not able to strictly purify both kind of vesicles leading to high variability between experiments.

Our data indicate that EV uptake is an active cellular process since no signal was detected neither when using Enduren-loaded fixed cells as targets nor when target cells were incubated with EVs at 4 °C (Fig. 4A). As a proof of concept that this method can serve as a robust platform for the screening of factors affecting the process of EV uptake, we treated receptor cells with small molecular inhibitors (Fig. 5B), which have been in many cases previously reported to impair EV uptake^23^. Our results support the idea that in SUM159 human breast cancer cells EV uptake is highly dependent on dynamin2 activity, whereas clathrin seems to be also involved, but to a lower extent. None of the inhibitors blocked completely EV uptake, supporting the notion that several overlapping routes of EV incorporation play a role in this process^23^. Interestingly, although it has been reported that heparin, EIPA and wortmannin can inhibit EV uptake^14,45^, we have not been able to detect any significant effect of these inhibitors at the dose and times analysed in our EV uptake assays with SUM159 cells.

## Conclusion

We provide here the basis for a luminiscence-based sensitive and quantitative assay to perform end-point assays and kinetics measurements at early stages of EV uptake, clearly discriminating from EV binding to the target cell. The assay works efficiently with small sample volumes, and measurements are fast and can be automatized, making it ideal for the high-throughput screen of drugs or blocking antibodies. Additionally, the same samples can be used also to track EVs intracellularly by confocal fluorescence microscopy, thus allowing to gain information about the subcellular localization and fate of uptaken EVs.

## Supplementary information

Supplementary Figures and Table
