# Supplementary material for: Development of a quantitative method to measure EV uptake
Source: Sci Rep. 2019 Jul 19;9:10522. doi: 10.1038/s41598-019-47023-9 (PMC6642168; doi:10.1038/s41598-019-47023-9)
Supplement: Supplementary file 1 — Supplementary Figures and Table [file 41598_2019_47023_MOESM1_ESM.pdf]

Development of a quantitative method to measure EV uptake.

Authors: Víctor Toribio[1], Sara Morales[2], Soraya López-Martín[1,2], Beatriz Cardeñes[3], Carlos Cabañas[3] and María Yáñez-Mó[1,2,3*].

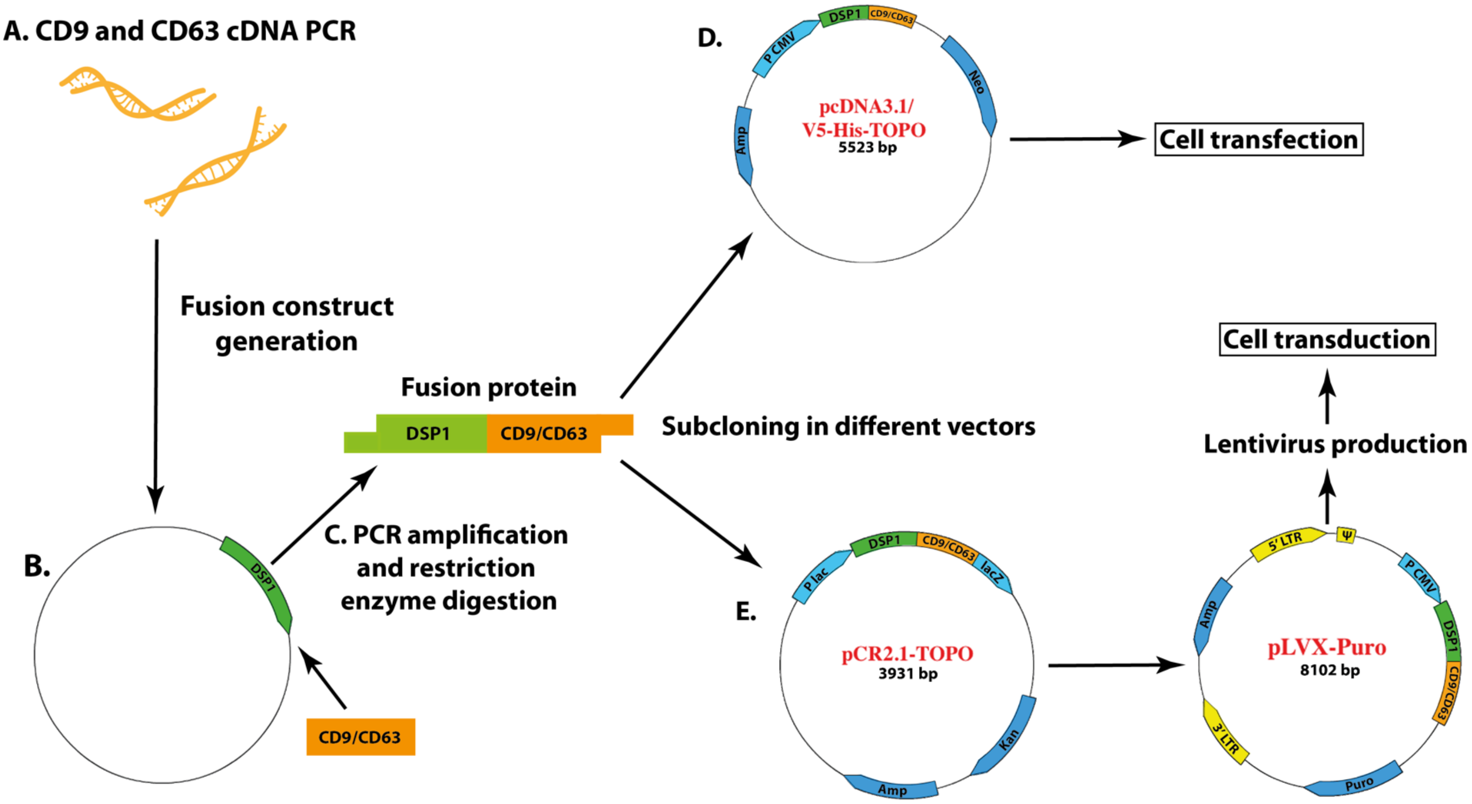

Supplementary Figure 1S. DSP1-CD9/CD63 fusion constructs design. A. CD9 and CD63 human cDNA sequences were amplified by PCR using specific oligonucleotides (**C**). Tetraspanin full coding sequence (CD9 or CD63) was inserted in reading frame after DSP1 C-terminus (**B**) **.** The fusion protein constructs, as well as DSP1 and DSP2 were amplified by PCR for subsequent cloning into two different vectors (**D** and **E**). **D.** DSP1-CD9/CD63 subcloning into pcDNA 3.3-TOPO vector allows for direct cell transfection. **E.** Subcloning into PCR 2.1-TOPO vector provided EcoR1 restriction sites for the final subcloning into pLVX-PURO lentiviral vector. HEK293 cells were transfected with pLVX-Puro vectors to produce lentiviral particles for viral transduction of cells with DSP1, DSP1-CD9/CD63 and DSP2.

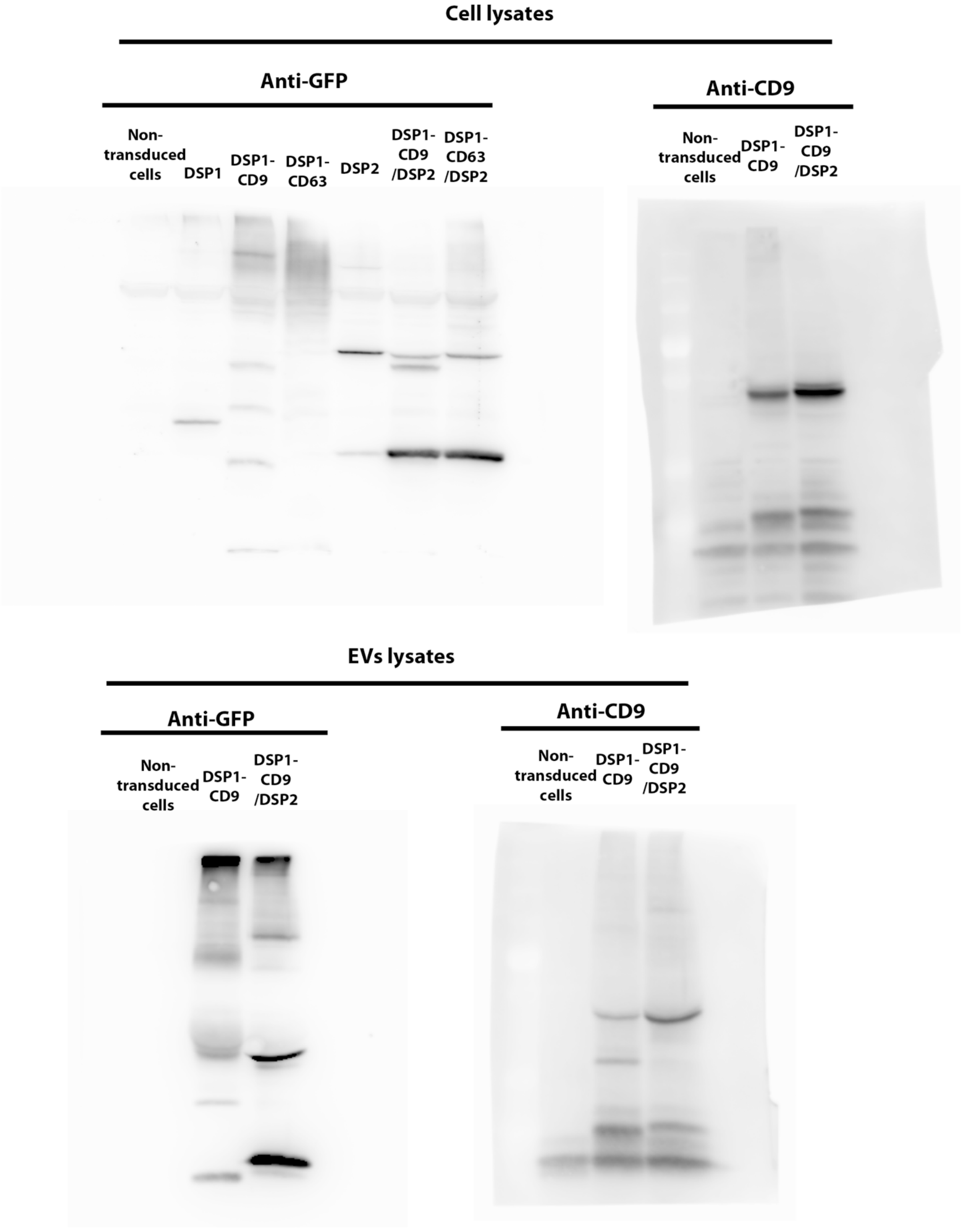

Supplementary Figure 2S. Original full-length gels of the western-blots shown in Figure 1D and E

	Interaction Anova Table				
**Figure**	**Sum of Squares**	**Degrees of Freedom**	**Mean Squares**	**F (DFn, DFd)**	**P value**
**Figure 3A**	1,18E+09	6	1,97E+08	F (6, 35) = 34.46	P < 0.0001
**Figure 3B**	6,89E+08	16	4,31E+07	F (16,36) = 9.72	P < 0.0001
**Figure 3C**	9,54E+08	12	7,95E+07	F (12, 48) = 118.7	P < 0.0001
**Figure 4A (left)**	8,67E+08	12	7,23E+07	F (12, 48) = 110.9	P < 0.0001
**Figure 4A (right)**	8,16E+09	24	3,40E+08	F (24, 54) = 15.63	P < 0.0001
**Figure 4B**	2,21E+10	24	9,20E+08	F (24, 78) = 6.506	P < 0.0001
**Figure 5A**	8,16E+09	24	3,40E+08	F (24, 54) = 15.46	P < 0.0001
**Figure 5B**	3,05E+01	20	1,52E+00	F (20, 64) = 24.91	P < 0.0001

Supplementary Table. Interaction, F-values and degrees of freedom of the ANOVA analyses from all graphs.
